# The Effects of Specific Omega-3 and Omega-6 Polyunsaturated Fatty Acids and Antioxidant Vitamins on Gait and Functional Capacity Parameters in Patients with Relapsing-Remitting Multiple Sclerosis

**DOI:** 10.3390/nu13103661

**Published:** 2021-10-19

**Authors:** Panayiotis Aristotelous, Manos Stefanakis, Marios Pantzaris, Constantinos S. Pattichis, Philip C. Calder, Ioannis S. Patrikios, Giorgos K. Sakkas, Christoforos D. Giannaki

**Affiliations:** 1 Department of Life and Health Sciences, University of Nicosia, Nicosia 2417, Cyprus; Aristotelous.panayiotis@gmail.com (P.A.); stefanakis.m@unic.ac.cy (M.S.); 2 The Cyprus Institute of Neurology and Genetics, Nicosia 2371, Cyprus; pantzari@cing.ac.cy; 3 Cyprus School of Molecular Medicine, Nicosia 2371, Cyprus; 4 Department of Computer Sciences, University of Cyprus, Nicosia 2109, Cyprus; pattichi@ucy.ac.cy; 5 Faculty of Medicine, University of Southampton, Southampton SO16 6YD, UK; P.C.Calder@soton.ac.uk; 6 NIHR Southampton Biomedical Research Centre, University Hospital Southampton NHS Foundation Trust and University of Southampton, Southampton SO16 6YD, UK; 7 School of Medicine, European University Cyprus, Nicosia 2404, Cyprus; i.patrikios@euc.ac.cy; 8 Department of PE and Sport Science, University of Thessaly, 42100 Trikala, Greece; gsakkas@pe.uth.gr; 9 School of Sports and Health Sciences, Cardiff Metropolitan University, Cardiff CF5 2YB, UK

**Keywords:** PUFA, relapsing remitting multiple sclerosis, gait, Neuroaspis^TM^ plp10, functional capacity

## Abstract

Patients with multiple sclerosis (MS) are characterized by, among other symptoms, impaired functional capacity and walking difficulties. Polyunsaturated fatty acids (PUFAs) have been found to improve MS patients’ clinical outcomes; however, their effect on other parameters associated with daily living activities need further investigation. The current study aimed to examine the effect of a 24-month supplementation with a cocktail dietary supplement formula, the Neuroaspis^TM^ PLP10, containing specific omega-3 and omega-6 PUFAs and specific antioxidant vitamins on gait and functional capacity parameters of patients with MS. Fifty-one relapsing-remitting MS (RRMS) patients with low disability scores (age: 38.4 ± 7.1 years; 30 female) were randomized 1:1 to receive either a 20 mL daily dose of the dietary formula containing a mixture of omega-3 and omega-6 PUFAs (12,150 mg), vitamin A (0.6 mg), vitamin E (22 mg), and γ-tocopherol (760 mg), the OMEGA group (*n =* 27; age: 39 ± 8.3 years), or 20 mL placebo containing virgin olive oil, the placebo group (*n =* 24; age: 37.8 ± 5.3 years). The mean ± SD (standard deviation) Expanded Disability Status Scale (EDSS) score for the placebo group was 2.36 and for the OMEGA group 2.22. All enrolled patients in the study were on Interferon-β treatment. Spatiotemporal gait parameters and gait deviation index (GDI) were assessed using a motion capture system. Functional capacity was examined using various functional tests such as the six-minute walk test (6MWT), two sit-to-stand tests (STS-5 and STS-60), and the Timed Up and Go test (TUG). Isometric handgrip strength was assessed by a dynamometer. Leg strength was assessed using an isokinetic dynamometer. All assessments were performed at baseline and at 12 and 24 months of supplementation. A total of 36 patients completed the study (18 from each group). Six patients from the placebo group and 9 patients from the OMEGA group dropped out from the study or were lost to follow-up. The dietary supplement significantly improved the single support time and the step and stride time (*p* < 0.05), both spatiotemporal gait parameters. In addition, while GDI of the placebo group decreased by about 10% at 24 months, it increased by about 4% in the OMEGA group (*p* < 0.05). Moreover, performance in the STS-60 test improved in the OMEGA group (*p* < 0.05) and there was a tendency for improvement in the 6MWT and TUG tests. Long-term supplementation with high dosages of omega-3 and omega-6 PUFAs (compared to previous published clinical studies using PUFAs) and specific antioxidant vitamins improved some functional capacity and gait parameters in RRMS patients.

## 1. Introduction

Multiple sclerosis (MS) is a progressive inflammatory and neurodegenerative disease of the central nervous system (CNS) [1]. MS is the leading neurological cause of disability in young adults, associated with many physical and mental impairments [2,3]. Several components contribute negatively to the quality of life (QoL) of patients with MS with a wide range of debilitating symptoms, including muscle weakness, fatigue, and balance and gait abnormalities, resulting in a significant financial burden on the patient, family, health system and society [4,5,6,7].

In order to maintain their independence, patients with MS must present adequate functional capacity [8]. Both QoL and independent living are strongly related to sufficient functionality, both in the upper and lower extremities [9,10]. Upper extremity motor function, coordination, and sensation are known to be affected in patients with MS, leading to damaging alterations, reduction in muscle function, and dependence on others for performing daily life activities [11,12] such as driving, drinking, eating, and writing [13]. Furthermore, inadequate lower extremity muscle function levels negatively influence essential activities such as stair climbing, balance, and walking [14,15].

Difficulty in walking is one of the most commonly reported problems in MS [16], as up to 93% of patients with MS report limitations in their walking 10 years following diagnosis. Interestingly, MS has a negative impact on gait despite the relatively low disability status [17] and presents major personal, social, and economic burdens on those living with MS [7].

Nutrition is commonly accepted as one of the possible environmental factors involved in the pathogenesis of MS, but the role of nutritional modulation or supplementation as a complementary MS treatment is unclear and largely disregarded [18,19]. Both specific omega-3 and omega-6 polyunsaturated fatty acids (PUFAs) and specific antioxidants are involved in nervous system function [20,21,22]. In 2013, Pantzaris et al. published the results of a randomized, double-blind, placebo-controlled clinical trial using a novel oral dietary supplement formula of specific omega-3 and omega-6 PUFAs and antioxidant vitamins compared with placebo. The results showed a significant effect in reducing the annual relapse rate as well as the progression of the disability with no-significant side effects [23]. Interestingly, the same supplement formula has been reported to significantly improve functional capacity and cognitive function in older people with mild cognitive impairment [24]. The scope of the current study is to investigate whether the same dietary formula, known as Neuroaspis^TM^ PLP10, containing a high dose of docosahexaenoic acid and eicosapentaenoic acid (DHA+EPA; omega-3 PUFAs) and linoleic acid and γ-linolenic acid (LA+GLA; omega-6 PUFAs) compared to the amounts used in previous clinical studies using PUFAs, and specific antioxidant vitamins (vitamin E as α-tocopherol and γ-tocopherol) can improve the functional capacity, muscle function, or gait, which can potentially contribute to the QoL and overall well-being, in patients with relapsing remitting multiple sclerosis (RRMS).

## 2. Materials and Methods

### 2.1. Participants

**Figure 1:**
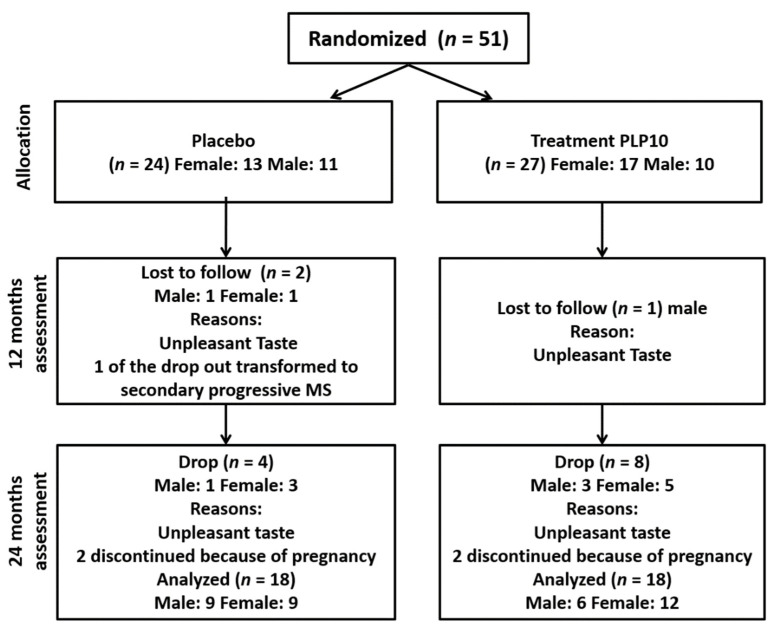
Flow of participants through the study.

Fifty-one patients with RRMS (age: 38.4 ± 7.1 years; 30 females) from the neurology clinics of the Cyprus Institute of Neurology and Genetics (CING) were voluntarily recruited into the study [25]. All of them were diagnosed according to the revised McDonald criteria 2011 [26]. The patients were randomized 1:1 into the OMEGA group (*n* = 27, 17 female) and the placebo group (*n* = 24, 13 female) (Figure 1). The patients’ characteristics are presented in Table 1. The inclusion criteria were: patients with relapsing-remitting MS that experienced at least one documented clinical relapse and with an Expanded Disability Scale Score (EDSS) of less than 5. The exclusion criteria were: patients with experience of a relapse within the last six months, with severely impaired visual function, with a severe psychiatric disorder, with severe arthritis of the knee or hips, pregnancy, with a significant medical condition (e.g., cancer or cerebrovascular disease), with any other neurological or vestibular disorders; and use of omega-3 and/or omega-6 and/or vitamin A or E supplementation or γ-tocopherol within the last 6 months. All participants were asked to retain their usual dietary habits during the study period. All participants gave written informed consent at the time of enrolment. The study was approved by the National Bioethics Committee (ΕΕΒΚ/ΕΠ/2013/18) and it was conducted in accordance with the Declaration of Helsinki.

Overall, six patients from the placebo group and nine from the OMEGA group dropped out of the study due to palatability issues (*n* = 10), pregnancy (*n* = 4), or changed from relapsing remitting to secondary progressive MS (*n* = 1) (see Figure 1). Thus, 36 patients completed the study, 18 patients (9 females) in the placebo group and 18 patients (12 females) in the OMEGA group (Figure 1). All the dropouts were excluded from the statistical analysis.

### 2.2. Randomization and Masking

An independent researcher performed the computer-generated 1:1 randomization. The investigators, research personnel, pharmacist, nursing and medical personnel, and participants were blinded to treatment allocation throughout the study. The supplement formulations had identical appearance and smell and were kept in dark bottles with coded numbers, blinded for both the participants and investigators. Participants were randomized to receive either a daily dose of a 20 mL cocktail formula (Neuroaspis^TM^ PLP10) consisting of omega-3 PUFAs ((EPA (810 mg) and DHA (4140 mg)), omega-6 PUFAs ((GLA (1800 mg) and LA (3150 mg)) (1:1 *w*/*w*), vitamin A (0.6 mg), vitamin E (22 mg as α-tocopherol) and γ-tocopherol (760 mg) plus citrus-aroma or 20 mL of placebo (pure virgin olive oil) with citrus-aroma for taste and palatability reasons [23,24]. The intervention and placebo were isocaloric. The supplements were consumed orally once daily, 30 min before dinner, using a dosage-calibrated cup, for 24 months following a 6 month pre-entry normalization period according to Pantzaris et al. [23]. The patients were instructed to keep the bottles in dark cool place or refrigerated. The pharmacist at CING was responsible for the appropriate storage and handling of the formulations including providing them to the participants. The adherence to the treatment was ensured by asking the patients to return the empty medication containers in order to receive replacements.

### 2.3. Procedures and Endpoints

Depending on their clinical status and in accordance with common practice, the participants continued to receive their regular treatment, with persistent evaluation for any side effects and adverse events. Clinical assessment visits were scheduled at baseline and at 12 and 24 months of the treatment period. The patients were also clinically examined by the treating neurologist within 48 hr after the onset of new or recurrent neurological symptoms [23]. The neurologist was trained to standardize the EDSS scoring procedures, examined the patients, made all medical decisions, determined the EDSS score, and reviewed the adverse or side effects. The patients were able to contact their neurologist at any time if there was any adverse event, side effect, or allergic reaction. The intervention was not expected to have any clinical or laboratory adverse effects different from those of the placebo that could disturb the double-blind nature of the trial. Therefore, the study neurologist functioned as both the treating and evaluating physician [23]. According to the previous studies no serious adverse effects were ever reported due to the formula and Neuroaspis^TM^ PLP10 is sold over the counter as a food supplement. All enrolled patients in the study were on Interferon-β treatment and Neuroaspis^TM^ PLP10 was used as supplement for the total study period.

The entire protocol followed the clinical trial guidelines as required by the USA Food and Drug Administration, European Medicines Agency, and the Committee for Medicinal Products for Human Use [23,27].

### 2.4. Anthropometry and Body Composition Assessment

Anthropometric characteristics including height and body weight were measured using a standing stadiometer (Seca model 220, Seca, Hamburg, Germany) and an analogue scale (Seca model 755, Seca, Hamburg, Germany), respectively. Total body fat was assessed by bioelectrical multi-frequency impedance analysis (Bodystat Quadscan 4000, Bodystat Ltd., Isle of Man, UK) with the subjects in the supine position. Current-injector electrodes were placed just below the metacarpo-phalangeal joint of the subjects in the middle of the dorsal side of the right hand and below the metatarsal arch on the superior side of the right foot. Detector electrodes were placed on the posterior side of the right wrist, midline to the pisiform bone of the medial (fifth phalangeal) side with the wrist semi flexed. Trunk fat percentage was quantified by the use of the BIA device ViScan (Tanita AB-140, Tanita Corp., Tokyo, Japan). A wireless ‘electrode belt’ was placed on the bare waist of the subject in a supine position.

### 2.5. Gait Analysis

A stereophotogrammetric system (Vicon Motion Systems Ltd., Oxford, UK) was used for the acquisition of the lower limb kinematics. Spatiotemporal and kinematic data acquired with eight infrared cameras and a Video Camera (DV1 Bonita 720c) at a sampling rate of 100 Hz using Vicon Nexus 2.8 software were also used. Knee and ankle joint width (mm) was measured in the weight-bearing position by the Abthroflex Small Bone Anthropometer Sliding Caliper. Leg length (mm) was measured from the anterior superior iliac to the ankle joint (medial malleoli) by the Abthroflex Anthropometric tape measure. Knee and ankle width and leg length were used in the scaling of the model. Participants had 14-mm^2^ passive reflective spherical markers placed on their lower limbs and the pelvis according to the Vicon Plug-In-Gait manual, based on the Helen Hays marker system [28], with two additional markers on each thigh and shin. The model used for capturing the kinematic variables was the PiG Lower Body Ai Functional 2 model which has the advantage that the center of rotation of the hip and the axis of rotation (flexion-extension) of the knee can be functionally calculated. The placement of markers (for thigh and tibia) is of little importance. Each patient performed six walking trials at their natural, comfortable self-selected speed in a 10 m walkway. All gait trials were performed in barefoot conditions, and the participants wore comfortable shorts (women also wore sports bras). On completion of gait analysis participants rested (by sitting down) for 10 min before commencing further testing. All data collection was carried out by the same investigator, experienced in analyzing gait. For every walking trial and each limb, spatiotemporal parameters (walking speed, cadence, stride time, stride length, stance phase, double support, step width etc.) were computed by the use of Vicon Polygon 4.2 software. The data were then averaged for each leg. Some gait parameters were normalized according to Hof et al. and Pinzone et al. [29,30], to facilitate comparisons between subjects with different anthropometric characteristics.

Gait Deviation Index (GDI) is a multivariate measure of overall gait quality [31]. GDI with a score of 100 indicates a subject without any neurological problem. Every 10 points that the GDI falls below 100 corresponds to one standard deviation away from the average unimpaired person. The GDI was computed automatically by the software using kinematic gait data from the pelvis, hip, knee, and ankle angles derived from the 3D gait analysis.

### 2.6. Functional Capacity Assessment

Patients’ functional capacity was assessed using a variety of tests commonly used in the MS population. In particular, the patients performed two sit-to-stand tests (STS-5 and STS-60) [32], the six-minute walk test [33] and the timed up and go test (TUG) [34]. The STS-5 requires the patients to perform five sit-to-stand cycles on a standard chair (0.43 m height and 0.45 m width) as fast as possible, measured in seconds, and can indicate the patient’s lower extremities strength [32]. The STS-60 requires patients to stand up and sit down to a chair as many times as possible in 60 s. The score is the total number of sit-to-stand cycles within the 60 s (the score achieved in 30 s was recorded as well) which is an index of muscular endurance. The six-minute walk test requires the patients to cover as much distance (in meters) within six minutes [32] and is considered an index of aerobic capacity. The TUG test requires the patients to rise from a chair, walk to a line on the floor 3 m away, turn around, walk back to the chair, and sit down again [34]. A shorter time indicates a better functional performance.

### 2.7. Handgrip Strength Assessment

A portable handgrip dynamometer (Takei, Tokyo, Japan) was used to measure the maximal handgrip strength (kg) of both hands. Participants were seated in a standard chair without armrests (seat height 0.43 m, seat width 0.45 m). They were instructed to sit in the middle of the chair, with their back straight, the shoulder in neutral position and the elbow in 90° flexion position holding the dynamometer, without touching the trunk. Three maximal effort trials lasting 4 to 5 s interspersed with 60 s rests were performed, and the highest value was retained for the analysis.

### 2.8. Isokinetic Dynamometry

Maximal voluntary strength of both legs was assessed using an isokinetic dynamometer (model 770, CSMI Humac Norm, Stoughton, MA, USA) which allowed the recording of instantaneous isokinetic torque. After a five min standardized warm-up on a bicycle, strength tests were performed on an adjustable chair (backwards inclined 5°) in a seated position. The alignment between the knee joint and the dynamometer rotational axis was adjusted to correspond to the femoral condyle axis, with the lever arm secured 10 cm above the ankle. All positional adjustment measures for each person were recorded for future use and standardization of the testing conditions. The upper leg, hip, and shoulders were secured to the equipment with safety straps [35]. The range of motion was set at 0–90° (0° corresponding to full knee extension). Before each test, individual calibration was completed for gravity correction [36]. This correction was determined at the position of 30° of knee flexion. During the trial, participants were instructed to keep the arms crossed with the hands on the opposite shoulder to isolate the quadriceps during the torque production [37]. Following 3 submaximal trial knee, isokinetic contractions and 1 min period of rest, bilateral isokinetic (concentric/concentric) flexion and extension of the right and left knee at 60°/s was performed for five times. The patients had periods of rest between the sessions, and the verbal encouragement was standardized. The highest of five isokinetic extension and flexion torques (Nm) selected as the peak dynamic torque and subsequently normalized to body weight. It is noteworthy to mention that all participants were able to perform the gait analysis and functional assessments without injuries and adverse effects.

### 2.9. Statistical Analysis

The sample size was estimated based on the previous proof-of-concept clinical trial [23]. Descriptive statistics were used to calculate the mean and standard deviation of spatiotemporal and kinematic variables. Independent sample T-tests were used to examine any differences between the two groups at baseline. Two-way (time X group) analysis of variance (ANOVA) with repeated measurements on both factors was used to examine the effect of treatment on the examined parameters. All analyses were performed using the Statistical Package for the Social Sciences software (SPSS for Windows, version 19.0, Chicago, Illinois). All data are presented as mean ± SD with the level for statistical significance set at *p* ≤ 0.05.

## 3. Results

### 3.1. Spatiotemporal Gait Parameters

A significant time X group interaction was found for left single support time, which was decreased in the OMEGA group and increased in the placebo group, respectively (*p =* 0.035) (Table 2). Moreover, there was a statistically significant time effect (*p =* 0.047) and time X group interaction (*p =* 0.025) regarding right step time, with a 2.1% increase in the placebo group and a 5.2% decrease in the OMEGA group after 24 months (Table 2). There was a marginally significant time group interaction (*p =* 0.051) for left stride time which was progressively, however slightly, increased in the placebo group (Table 2). In the OMEGA group, there was an increase in left stride time from 1.12 s at baseline to 1.14 s at 12 months and then a decrease to 1.05 s at 24 months. There was however no statistically significant time (*p =* 0.148) or group effect (*p* = 0.073).

There was a statistically significant group X time interaction (*p* = 0.039) but no effect of time (*p =* 0.196) or group (*p =* 0.060) with regards to right stride time (Table 2). Briefly, right stride time was progressively increased in the placebo group from 1.15 s at baseline to 1.16 s at 12 months and finally to 1.17 s at 24 months. In the OMEGA group there was an increase in right stride time from 1.11 s at baseline to 1.13 s at 12 months and then a decrease to 1.04 s at 24 months (Table 2). In the 24 months assessment, for the placebo group, there was an 1.7% increase in right stride time from baseline while in the OMEGA group there was a 6.3% decrease.

At 24 months, left leg GDI was decreased by 10.5% in the placebo group while in the OMEGA group was increased by 3.4% (Table 3). The results revealed a statistically significant interaction between group and time (*p =* 0.001), a statistically significant time effect (*p =* 0.028) between the baseline and the 24-month follow up assessment but no statistically significant group effect (*p =* 0.233).

At 24 months, the right GDI was decreased by 9.5% in the placebo group while in the OMEGA group it was increased by 4.3%. The results revealed a statistically significant interaction between group and time (*p =* 0.001), but no statistically significant time (*p =* 0.185) or group effect (*p =* 0.075).

The results did not reveal any statistically significant time effect (*p =* 0.097) or interaction between group and time (*p =* 0.110) with regards to left leg walking speed. There was however a statistically significant group effect (*p =* 0.024). Similarly, the results did not reveal any statistically significant time effect (*p =* 0.149) or interaction between group and time (*p =* 0.140) with regards to right leg walking speed, but a statistically significant group effect (*p =* 0.024) (Table 2). In the normalized cadence of the left leg there was no time (*p =* 0.259) or group (*p =* 0.154) effect or interaction between group and time (*p =* 0.099) (Table 2). With regards to normalized cadence of the right leg, the results revealed a statistically significant time effect (*p =* 0.001), but no statistically significant group effect (*p =* 0.098) or interaction between group and time (*p =* 0.069).

### 3.2. Functional Capacity Assessment

With regard to the 6MWT, the participants of the placebo group increased the average walking distance from 504 m at baseline to 523 in the 12 month assessment but then it dropped to 494 m at the 24 months follow up (Table 3). For the OMEGA group, the patients showed a progressive increase in the average walking distance from 505 m at baseline to 546 m at the 12 months and finally to 551 m at 24 months (Table 3). For the 24 month 6MWT assessment, the average distance decreased by 2% in the placebo group and increased by 9.1% in the OMEGA group. For the 12 month assessment the average walking distance increased by 3.7% in the placebo group and by 7.7% in the OMEGA group. Between the 12 and 24 month follow-up, 6MWT distance decreased by 5.5% in the placebo group and increased by 0.9% in the OMEGA group. Despite these observations, there was no time (*p =* 0.137) or group (*p =* 0.186) effect for the 6MWT and no statistically significant interaction between group and time (*p =* 0.337) (Table 4).

Performance on the STS-5 test was decreased in both groups after the 24 month intervention period. In particular, for the placebo group the STS-5 time decreased from 12.5 s at baseline to 10.6 s at 12 months and then increased to 11.5 s at 24 months (Table 4). For the OMEGA group, the STS-5 time was progressively decreased from 12.3 s at baseline to 10.3 s at 12 months and finally to 9.9 s at 24 months (Table 4). At 24 months STS-5 time improved from baseline in the placebo group by 8.3% and in the OMEGA group by 19.9%. Between the baseline and the 12 month follow up assessment as well as between the baseline and the 24 month follow up assessment there was a statistically significant time effect (*p =* 0.001). However, no statistically significant group effect (*p =* 0.359) was identified, whilst there was a trend for a significant time X group interaction (*p =* 0.078).

For the placebo group, the 24 month STS-30 test was improved by 5.3% from baseline and for the OMEGA group it was improved by 22.3% (Table 4). A statistically significant time effect (*p =* 0.001) was recorded between the baseline and 12 month follow up and between the baseline and 24 month follow up. Additionally, there was a statistically significant interaction between group and time (*p =* 0.040). No statistically significant group effect was observed (*p =* 0.297).

STS-60 was improved at 24 months by 4.9% in the placebo group and by 20.7% in the OMEGA group (Table 4). A statistically significant time effect (*p =* 0.001) was identified between the baseline and 12 month follow-up and between the baseline and 24 month follow-up, as well as a statistically significant interaction between group and time (*p =* 0.032). No statistically significant group effect was recorded (*p =* 0.279).

The TUG time for the 24 month assessment in the placebo group was improved by 1.1% from baseline and in the OMEGA group it was improved by 7.1% (Table 4). No statistically significant group effect (*p =* 0.097) or interaction between group and time was observed (*p =* 0.411). There was a significant time effect between the baseline and the 24 month follow up (*p =* 0.003).

There was no statistically significant time effect, or group effect or interaction between group and time for body fat, trunk fat or abdominal girth (Table 5).

### 3.3. Handgrip Strength

The peak grip strength of the right hand was increased by 6.6% in the placebo group during the first 12 months of the study while in the OMEGA group it was increased by 3.4% (Table 6). From the 12 month to the 24 month assessment the placebo and OMEGA groups showed improvement by 6.5% and 10.5%, respectively. At 24 months the grip strength of the right hand was increased by 12.7% in the placebo group and by 14.1% in the OMEGA group (Table 6). No statistically significant group effect (*p* = 0.796) or interaction between group and time (*p =* 0.739) was recorded. A significant time effect was found between baseline and 12 months and between 12 and 24 months (*p =* 0.001). Similar findings were observed for left handgrip strength (Table 6). During the first 12 months, the peak left handgrip strength was improved by 3.5% in the placebo group and by 8.1% in the OMEGA group. Between 12 and 24 months, the left handgrip strength was improved for both groups, by 1.8% and 5.8% respectively (Table 6). At 24 months the average strength was improvement from baseline was 5.4% in the placebo group and 14.3% in the OMEGA group without any statistically significant group effect (*p =* 0.560) or interaction between group and time (*p =* 0.415). A statistically significant time effect was found between the baseline and the 24 month follow up (*p =* 0.012).

### 3.4. Isokinetic Knee Strength

The maximum average right knee extension strength was decreased in the placebo group by 4.5% during the first 12 months of the study, while in the OMEGA group it was improved by 4.5% (Table 5). From 12 to 24 months, the right knee extension strength was improved in both groups, by 3.1% in the placebo group and by 0.8% in the OMEGA group. At 24 months for the patients in the placebo group the right knee extension strength was decreased by 1.4% while for the patients in the OMEGA group it was increased by 5.5%. There was no statistically significant time effect (*p =* 0.774) or group effect (*p =* 0.255), or a statistically significant interaction between group and time (*p =* 0.421) (Table 6).

During the first 12 months of the study the maximum left knee extension strength was decreased by 0.5% in the placebo group but for the OMEGA group it was increased by 3.2% (Table 6). From 12 to 24 months the left knee extension strength was increased for both groups, by 11.2% for the placebo group and by 3.6% for the OMEGA group. From baseline to 24 months the left knee extension strength was improved by 10.7% in the placebo group and by 7% in the OMEGA group (Table 6). A statistically significant time effect (*p =* 0.037) between the baseline and the 24-month follow-up was identified.

For both legs there was a statistically significant time effect (*p =* 0.025) between the baseline and the 12 month assessment, between the baseline and the 24 month assessment and between the 12 and the 24 month assessment. No statistically significant group effect (*p =* 0.484) or interaction between group and time (*p =* 0.373) was observed (Table 6).

Over 24 months, the right knee flexion strength was reduced by 25.9% in the placebo group and by 21.1% in the OMEGA group, and the left knee flexion strength was reduced by 23.6% and 22.8% in the two groups, respectively (Table 6).

## 4. Discussion

MS is a chronic multifactorial inflammatory disease, where several different biochemical processes simultaneously contribute to the pathogenesis. This study aimed to examine the effects of a 24-month dietary supplementation with a cocktail formula containing a high dose of specific omega-3 (EPA+DHA) and omega-6 (LA+GLA) PUFAs along with specific antioxidant vitamins, including vitamin E and γ-tocopherol (Neuroaspis^TM^ PLP10) on gait and functional capacity parameters in patients with RRMS. The supplement is designed to target inflammatory and oxidative stress processes, but may have other effects directly on neuronal function and on skeletal muscle. These effects together might improve the outcomes under study here. Indeed, the supplement was found to improve a number of the parameters under investigation (including functional capacity and gait of the patients). This novel approach could potentially be crucial in improving the QoL of patients with RRMS.

MS results in significant negative alterations in gait despite the relatively low disability status [17], and presents major personal, social, and economic burdens for those living with MS [7]. MS patients display slower walking speed, reduced stride length [38], prolonged double limb support [39] with fewer, shorter, and wider steps [40] compared to their matched healthy controls. People select a specific walking speed [41] that minimizes the energy expenditure per unit distance [41] and any walking speed, other than their particular walking speed, significantly affects kinematic and kinetic gait patterns [42]. MS patients with moderate disability walk slower with shorter step length, alterations which elevate the energetic cost of walking [43]. Furthermore, MS patients often experience falls due to negative alterations in balance and mobility [44]. Falls in MS patients are related to injuries, activity limitation, and further deterioration in mobility levels [45,46] with a consequent impact on patients’ QoL [47].

In the current study the Neuroaspis^TM^ PLP10 dietary supplement has been shown to improve spatiotemporal parameters, such as the left single support time, the right step time, and the stride time of both legs. On the other hand, there was no significant improvement of other parameters such as cadence and walking speed. The lack of improvement in these parameters can be attributed to the fact that the participants in this study had low disability status and thus the margin for improvement was small and not easily achieved with a small sample size. It is noteworthy that the literature does not provide a clear minimal clinically significant difference on spatiotemporal parameters [17]. Moreover, the studies in the literature mostly evaluate patients with MS with much higher level of disability than we studied [37,46], thus having a larger possibility for spatiotemporal parameters improvement. Even though a limited deviation from normal gait was observed, a small improvement was recorded, with promising positive changes in the spatiotemporal parameters.

The GDI is a multivariate measure of overall gait quality. It is computed with kinematic gait data from the pelvis, hip, knee, ankle, and foot. It is an index that measures the distance between any chosen gait vector and a gait vector averaged over a control group [31]. GDI showed some considerable group by time interaction. Precisely, while GDI of the control group between the baseline and the 24 month follow up decreased across legs by about 10% on average, the opposite was found for the OMEGA group, where GDI increased by about 4% on average across both legs. These results suggest that the dietary supplement might have a significant protective effect against overall deterioration of gait.

Functional capacity is considered to be a highly important factor for the QoL and for the overall wellbeing of patients with MS. The findings of the current study suggest that a 24 month supplementation with the aforementioned dietary formula could improve performance based on various functional capacity tests, such as the sit-to-stand tests, as well. Moreover, the effects on walking performance appeared to be promising but without statistically significant changes.

The 6MWT generally provides an indirect assessment of walking fatigability, an estimation of maximal walking distance, a measure of functional capacity [48], and a prediction in changes in everyday activities such as habitual walking [49] in MS patients. The results of this study showed no statistically significant difference between the two groups for the 6MWT distance. However, while the placebo group reduced the distance of 6MWT at the 24 month follow up, the OMEGA group increased the distance by 46 m. According to the study of Baert et al., clinically meaningful changes from the patient’s perspective may be 21.6 m on the 6MWT for MS patients [50]. Therefore, the increase in the walking distance in the OMEGA group, although not statistically significant different vs the control group, could be considered clinically significant. Furthermore, the results of the 6MWT are in accordance with the results of the GDI and further support the positive effect of the supplement in gait.

The ability to undertake STS movements is considered an essential feature for determining the degree of independence and the QoL of a person [51]. In the hierarchy of disability, problems in standing up manifest much later than limitations in walking [52]. The current study revealed that the nutritional supplement significantly improved the performance of patients in the STS assessments compared with placebo. Considering the fact that STS tests are considered mechanically demanding movements in relation to the daily living activities [53,54,55], specifically due to the high level of muscle activation requirements (since an individual needs to coordinate a transfer from a horizontal to a vertical position in a single movement), the positive effects on this parameter can be considered clinically and practically important [56]. In agreement with the findings of this study, Rodacki et al. reported that fish oil supplementation can improve the outcome of STS tests [57]. Thus, the benefit of the supplement on this outcome may relate to its content of the omega-3 PUFAs, EPA, and DHA.

In lower extremity muscle assessment, both placebo and OMEGA groups had similar knee extensors strength in the isokinetic evaluation at 24 months, with the knee flexors strength reduced. Although adequate extensors muscle strength is mainly required for the integration of the STS movement, since both groups displayed near identical extensor strength, this is probably not responsible for the improvement in the STS performance of the OMEGA group.

Speculatively, the trend for improvement in a number of tests (e.g., GDI, 6MWT, TUG) in the OMEGA group vs. placebo group for parameters related to motor control, stability, and mobility could be due to an improvement in postural control or muscle fatigue.

Handgrip strength is an important predictor of poorer health status, and an impairment in grip strength is a contributing factor of worsening QoL for MS patients [12]. The results for the handgrip strength assessment showed that both groups improved their strength during the 24 month intervention period with no statistically significant difference between them. This result was unexpected, since MS is a progressive disease impairing motor function affecting handgrip strength. It is worth noting that the participants in the study were not participating in any exercise or physical activity programs during the trial and thus, no strengthening improvement across either group was anticipated. It may be relevant that the disability status of the participating MS patients was low at baseline.

Lower body muscle strength is considered as an important predictor of ambulatory function [58]. Individuals with MS have significantly reduced muscle strength, and the ability to generate maximal force [59] in the lower extremities is reduced compared to healthy controls [60]. The results indicated that both groups increased their extension strength during study. Meanwhile, the knee flexion strength was reduced. This suggests that the ratio of knee extensor to knee flexor strength increased which might account for the improvement in the functional tests such as STS.

### Limitations

The walking speed evaluated in this study was the natural self-selected speed of the subjects. The preferred self-selected speed was chosen as a means to evaluate subjects in the condition they spent most of their time in, during activities of daily living. An enforced higher walking speed is more likely to reveal differences between groups. Future studies should take this into consideration and assess both the self-selected speed as well as a higher more challenging speed, providing an opportunity to detect more subtle differences between groups and over time. Moreover, in order to complete the STS movement, a variety of muscle groups need to be activated during the required movements. For this study, only the maximum strength of knee extensors and flexors was measured by the isokinetic dynamometer. The activity in the trunk (erector spinae, rectus abdominus), ankle (dorsi and plantar flexion), knee (quads and hamstrings) and hip muscles was not measured during the functional tests by an EMG; this is a limitation that affects the interpretation of some of the results. The small sample size was also a limitation of the present study. On the other hand, the length of the study (24 months), the range of tests used and the thorough statistical data analyses are all strengths and contribute to our understanding of this patient population and the design of further studies.

## 5. Conclusions

The results of the present study support the hypothesis that this specific dietary formula (Neuroaspis^®^ PLP10), a mixture of specific bioactive molecules, the omega-3 PUFAs DHA and EPA, the omega-6 PUFAs LA and GLA, and several antioxidant vitamins including vitamin E (α-tocopherol) and γ-tocopherol in a high dose, can act protectively against functional deterioration of patients with a progressive disease such as RRMS. Future studies should examine the effectiveness of this supplement in larger samples of patients as well as in MS patients with higher levels of disability. Furthermore, it will be valuable to study the combination of the supplement with simultaneous exercise in this group of patients.

## Figures and Tables

**Table 1:** Patient demographic and baseline characteristics.

Variables	Placebo Group	OMEGA Group	*p* Value
*N*	18	18	-
Age (years)	38.1 ± 5.3	39.1 ± 8.7	0.665
Weight (kg)	70.2 ± 17.2	70.1 ± 15.8	0.987
Height (cm)	166.7 ± 8.2	166.4 ± 8.9	0.923
Body mass index	25.1 ± 5.3	25.1 ± 4.3	0.997
EDSS score	2.36 ± 1.09	2.22 ± 1.08	0.705

Abbreviations: EDSS, Expanded Disability Scale Score. Data expressed as mean ± SD.

**Table 2:** Normalized spatiotemporal gait parameters of the left and right leg in both groups for all three time-points, excluding the dropouts.

Variables	Placebo Group (*n* = 18)	OMEGA Group (*n* = 18)	Main Effects and Interactions (*p* Value)		
			Time	Time X Group	Group
Left Single Support (s)					
Baseline	0.436 ± 0.04	0.440 ± 0.04	0.896	**0.035**	0.328
12 months	0.443 ± 0.03	0.437 ± 0.04			
24 months	0.452 ± 0.04	0.421 ± 0.03			
Right Step Time (s)					
Baseline	0.577 ± 0.06	0.555 ± 0.05	**0.047**	**0.025**	0.131
12 months	0.586 ± 0.06	0.579 ± 0.08			
24 months	0.589 ± 0.08	0.526 ± 0.05			
Left Stride Time (s)					
Baseline	1.16 ± 0.14	1.12 ± 0.09	0.148	**0.051**	0.073
12 months	1.17 ± 0.1	1.14 ± 0.16			
24 months	1.18 ± 0.15	1.05 ± 0.12			
Right Stride Time (s)					
Baseline	1.15 ± 0.14	1.11 ± 0.09	0.196	**0.039**	0.060
12 months	1.16 ± 0.1	1.13 ± 0.13			
24 months	1.17 ± 0.16	1.04 ± 0.11			

Data expressed as mean ± SD. Bold text indicates a statistically significant difference with a *p*-value less than 0.05.

**Table 3:** Gait Deviation Index, cadence and walking speed data in both groups for all three time-points, excluding the dropouts.

Variables	Placebo Group (*n* = 18)	OMEGA Group (*n* =18)	Main Effects and Interactions (*p* Value)		
			Time	Time X Group	Group
Left Gait Deviation Index					
Baseline	80.88 ± 12.61	78.38 ± 7.07	**0.028**	**0.001**	0.233
12 months	74.70 ± 10.08	79.20 ± 6.82			
24 months	72.38 ± 11.83	81.04 ± 7.87			
Right Gait Deviation Index					
Baseline	81.63 ± 9.92	81.80 ± 8.78	0.185	**0.001**	0.075
12 months	78.56 ± 9.63	82.40 ± 7.37			
24 months	73.82 ± 11.67	85.34 ± 7.76			
Normalized left cadence					
Baseline	31.15 ± 3.97	31.72 ± 2.64	0.259	0.099	0.154
12 months	30.49 ± 2.81	31.80 ± 3.82			
24 months	30.59 ± 3.79	33.97 ± 4.47			
Normalized right cadence					
Baseline	29.03 ± 0.84	29.03 ± 0.98	**0.001**	0.069	0.098
12 months	30.80 ± 0.84	32.22 ± 0.98			
24 months	30.81 ± 2.77	33.51 ± 3.70			
Normalized left walking speed					
Baseline	0.359 ± 0.61	0.402 ± 0.45	0.097	0.110	**0.024**
12 months	0.376 ± 0.06	0.399 ± 0.05			
24 months	0.370 ± 0.06	0.428 ± 0.06			
Normalized right walking speed					
Baseline	0.364 ± 0.06	0.402 ± 0.04	0.149	0.140	**0.024**
12 months	0.38 ± 0.06	0.408 ± 0.05			
24 months	0.369 ± 0.06	0.431 ± 0.06			

Data expressed as mean ± SD. Bold text indicates a statistically significant difference with a *p*-value less than 0.05.

**Table 4:** Functional capacity and disability score in both groups for all three time-points excluding the dropouts.

Variables	Placebo Group (*n* = 18)	OMEGA Group (*n* = 18)	Main Effects and Interactions (*p* Value)		
			Time	Time X Group	Group
Six Minute Walk Test Normalized (m)					
Baseline	504 ± 131	505 ± 99	0.137	0.186	0.337
12 months	523 ± 90	546 ± 73			
24 months	494 ± 79	551 ± 101			
STS5 (s)					
Baseline	12.57 ± 3.52	12.37 ± 2.83	**0.001**	0.078	0.359
12 months	10.67 ± 2.75	10.33 ± 2.26			
24 months	11.52 ± 1.97	9.91 ± 1.72			
STS 30 (rep)					
Baseline	12.55 ± 2.97	12.44 ± 2.63	**0.001**	**0.040**	0.297
12 months	14.66 ± 3.1	15.16 ± 2.66			
24 months	13.22 ± 2.18	15.22 ± 2.39			
STS60 (rep)					
Baseline	25 ± 6.10	25.16 ± 5.51	**0.001**	**0.032**	0.279
12 months	29.55 ± 6.20	30.33 ± 5.12			
24 months	26.22 ± 4.27	30.38 ± 4.86			
TUG (s)					
Baseline	8.90 ± 1.76	8.21 ± 1.47	**0.003**	0.411	0.097
12 months	8.04 ± 1.94	7.44 ± 1.84			
24 months	8.80 ± 1.61	7.63 ± 1.09			
EDSS					
Baseline	2.36 ± 1.09	2.22 ± 1.08	**0.001**	**0.001**	0.446
12 months	2.41 ± 1.10	2.27 ± 1.04			
24 months	2.83 ± 1.20	2.27 ± 1.04			

Abbreviations: STS-5, sit-to-stand test 5-repetitions; STS-30, sit-to-stand test 30 s; STS-60, sit-to-stand test 60 s; TUG, timed up and go test; EDSS, Expanded Disability Scale Score. Data expressed as mean ± SD. Bold text indicates a statistically significant difference with a *p*-value less than 0.05.

**Table 5:** Body composition, abdominal girth and trunk fat in both groups for all three time-points excluding the dropouts.

Variables	Placebo Group (*n* = 18)	OMEGA Group (*n* = 18)	Main Effects and Interactions (*p* Value)		
			Time	Time X Group	Group
Body Fat (%)					
Baseline	28.98 ± 9.16	29.36 ± 7.02	0.538	0.635	0.641
12 months	27.65 ± 10.44	29.20 ± 8.70			
24 months	27.80 ± 8.40	29.26 ± 8.08			
Abdominal girth (cm)					
Baseline	96.94 ± 18.93	93.61 ± 12.41	0.283	0.489	0.661
12 months	95.16 ± 17.05	92.94 ± 13.07			
24 months	94.11 ± 15.72	93.16 ± 12.39			
Trunk fat (%)					
Baseline	33.11 ± 12.94	32.07 ± 9.36	0.774	0.421	0.255
12 months	33.06 ± 12.32	31.62 ± 8.67			
24 months	31.37 ± 11.73	31.76 ± 9.50			
Body mass index					
Baseline	25.11 ± 5.37	25.11 ± 4.36	0.453	0.141	0.837
12 months	24.76 ± 5.19	24.98 ± 4.74			
24 months	24.58 ± 5.11	24.98 ± 4.70			

Results expressed as mean ± SD.

**Table 6:** Isometric handgrip strength and unilateral leg strength in both groups for all three time-points excluding the dropouts.

Variables	Placebo Group (*n* = 18)	OMEGA Group (*n* = 18)	Main Effects and Interactions (*p* Value)		
			Time	Time X Group	Group
Right Handgrip Strength (kg)					
Baseline	28.90 ± 9.60	28.24 ± 7.47	**0.001**	0.739	0.796
12 months	30.81 ± 11.44	29.25 ± 7.87			
24 months	32.58 ± 9.83	32.22 ± 8.87			
Left Handgrip Strength (kg)					
Baseline	29.33 ± 9.55	26.30 ± 6.69	**0.012**	0.415	0.560
12 months	30.36 ± 11.16	28.42 ± 7.02			
24 months	30.91 ± 11.89	30.07 ± 7.60			
Max Isokinetic Strength of Right Knee Extensors at 60°/s Normalized by BW (N·m/kg)					
Baseline	2.05 ± 0.79	2.19 ± 0.62	0.774	0.421	0.255
12 months	1.96 ± 0.89	2.29 ± 0.68			
24 months	2.02 ± 0.74	2.31 ± 0.42			
Max Isokinetic Strength of Left Knee Extensors at 60°/s Normalized by BW (N·m/kg)					
Baseline	1.95 ± 0.62	2.15 ± 0.45	**0.037**	0.618	0.284
12 months	1.94 ± 0.82	2.22 ± 0.59			
24 months	2.16 ± 0.74	2.30 ± 0.40			
Max Isokinetic Strength of Right Knee Flexors at 60°/s Normalized by BW (N·m/kg)					
Baseline	1.31 ± 0.45	1.42 ± 0.35	**0.001**	0.718	0.217
12 months	1.30 ± 0.55	1.50 ± 0.31			
24 months	0.97 ± 0.39	1.12 ± 0.28			
Max Isokinetic Strength of Left Knee Flexors at 60°/s Normalized by BW(N·m/kg)					
Baseline	1.31 ± 0.36	1.36 ± 0.31	**0.025**	0.373	0.484
12 months	1.24 ± 0.50	1.39 ± 0.36			
24 months	1.0 ± 0.46	1.05 ± 0.26			

Abbreviations: BW, Body weight. Data expressed as mean ± SD. Bold text indicates a statistically significant difference with a *p*-value less than 0.05.

## Data Availability

Not applicable.
